# Quantitative analysis of the morphing wing mechanism of raptors: Bionic design of Falco Peregrinus wing skeleton

**DOI:** 10.1371/journal.pone.0299982

**Published:** 2024-04-02

**Authors:** Di Tang, Wenxi Shi, Dawei Liu, Yin Yang, Liwen Zhu, Lang Xu

**Affiliations:** 1 College of Mechanical Engineering, Zhejiang University of Technology, Hangzhou, Zhejiang, China; 2 High Speed Aerodynamic Institute, China Aerodynamics Research and Development Center, Mianyang, Sichuan, China; Nasarawa State University, NIGERIA

## Abstract

The wing is one of the most important parts of a bird’s locomotor system and is the inspiration origination for bionic wing design. During wing motions, the wing shape is closely related to the rotation angles of wing bones. Therefore, the research on the law of bone movement in the process of wing movement can be good guidance for the design of the bionic morphing wing. In this paper, the skeletal posture of the peregrine falcon wing during the extension/flexion is studied to obtain critical data on skeletal posture. Since an elbow joint and a wrist joint rotate correlatively to drive a wing to flex/extend, the wing skeleton is simplified as a four-bar mechanism in this paper. The degree of reproduction of wing skeleton postures was quantitatively analyzed using the four-bar mechanism model, and the bionic wing skeleton was designed. It is found that the wing motions have been reproduced with high precision.

## Introduction

Flying birds in nature can use flapping wings to gracefully and dexterously traverse terrestrial, aerial, and aquatic environments. As concluded in our previous research, the key to achieving high manoeuvrability in bird flight lies not in the static aerodynamic performances but in how the wing morphs to change the flying situations [1,2]. Thus, it is the prerequisite to figure out how the bird morphs their wings.

In the last decade, the interest in morphing wing technology has gained a great deal of attention in aviation due to the superior benefits it can provide. Morphing wings, inspired by the long-term investigation of avian flight in nature, can change and optimize their wing shapes to adapt to various flight conditions and mission profiles, unlike traditionally fixed wings which merely focus on some specific flight conditions. In nature, birds obtained the remarkable ability to fly through long-term biological evolution [1]. For instance, they can take advantage of dynamic adjustment of wing postures to meet the requirements of different flying missions (e.g., efficient cruise, aggressive maneuver, and precision descents) [3]. The unique morphing structure of birds allows them to fold their wings into different swept-wing configurations dynamically, which inherently alters some important geometrical parameters such as wing area, wingspan, swept angle, chord length, and so on, improving aerodynamic efficiency and high-speed maneuverability at a diverse range of flight conditions. For example, black vultures change their wing spans by deploying and folding their primary feathers to perform two different behaviors effectively, including soaring and gliding [4]. Swifts accurately adjust sink speed and turning rate by modifying wing sweep during maneuvers [5]. Eagles actively sweep their wings back in response to turbulence [6,7]. Some other researchers also found that morphing swept wings can effectively improve yawing stability and pitch control during the flight in birds [8].

The advancement in investigations into avian flight has promoted the application of bio-inspired morphing wing technologies on aircraft. The current design of morphing wings [9–14] can be roughly classified into two major categories based upon morphing motion characteristics: 1) multi-rigid-body motion. 2) elastic skin deformation supported by a morphing skeleton structure. The multi-joint swept wing, whose inboard and outboard sweep angles can vary continuously by using two joint actuation points, can be regarded as a typical example of the morphing wing with the first motion characteristics [9]. The numerical simulation results show that the multi-joint swept wing could indeed improve the stability and maneuverability to some extent. Another example is given by the telescoping wing developed by Blondeau et al [10]. It is actuated by utilizing a pneumatic piston system and can change the wing area and wingspan simultaneously. There are several examples of the second morphing approach, for example, the Morphing Flight-vehicle Experimental (MFX-1) developed by NextGen Aeronautics [11].

**Fig 1:**
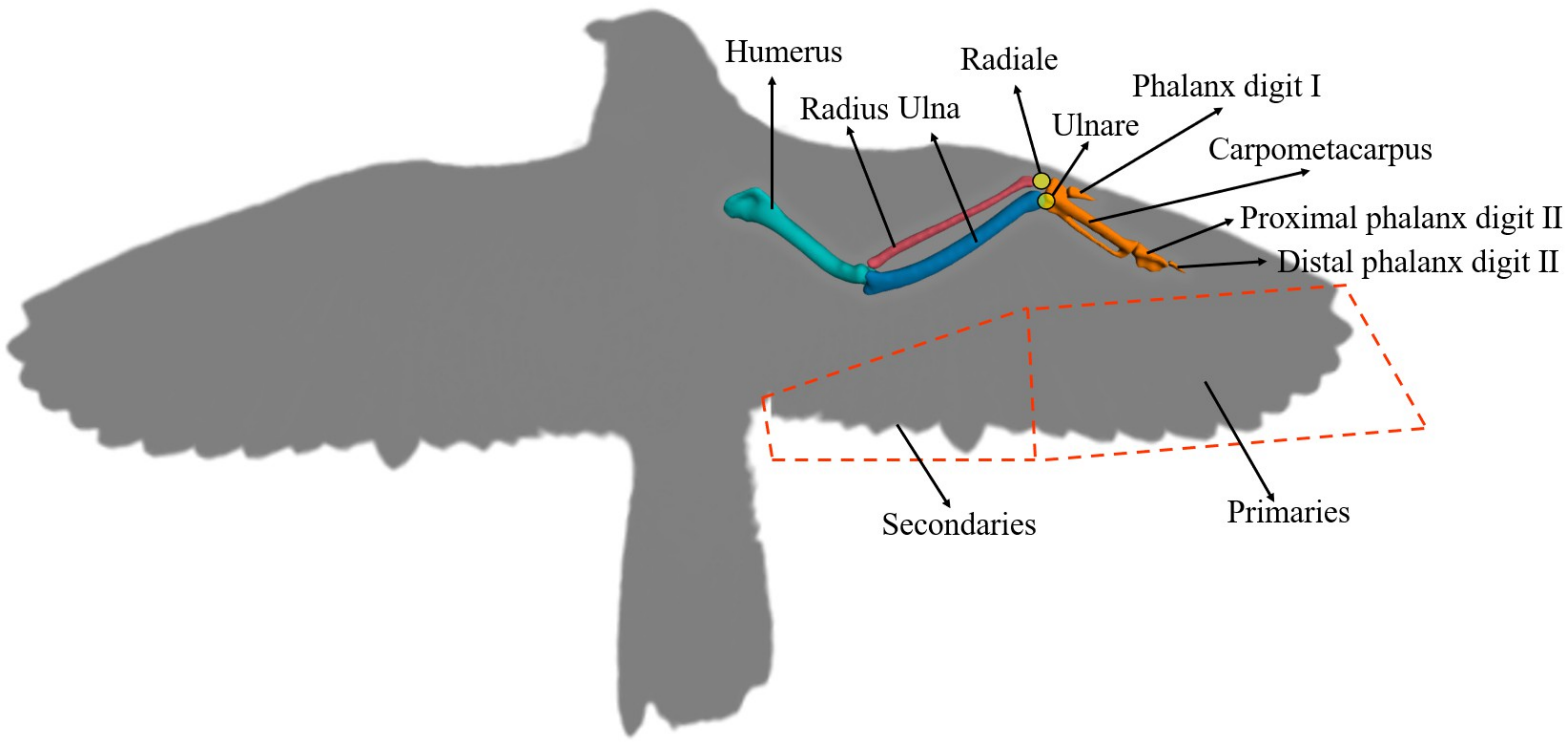
Peregrine falcon wing skeletal structure [19].

To obtain insight into the twist—swing mechanism of a bird wing, it is necessary to analyze the wing’s physiological structure. As shown in Fig 1, a bird wing is mainly composed of a skeleton wrapped by the skeletal muscle, alula, primaries, and secondaries [15]. The wing skeleton [see Fig 1] consists of the humerus, radius, ulna, metacarpal, phalanges, and some joints. Although the distance from the shoulder to the phalanges is less than half of the wing span, the wing skeleton undertakes all the aerodynamic force generated by the wing. Primaries and secondaries form the main contour of the wing [16]. The primaries are fixed to the metacarpal and phalanges through connective tissue, and the secondaries are fixed to the ulna through connective tissue. Tendons connect the two groups of feathers along the upper surface of the patagium and control the extension of the feathers as the elbow and wrist extend. The gap between the secondaries and the body is filled with feathers [not shown in Fig 1]. Primaries and secondaries are the main parts for generating aerodynamic force, and they transmit force and torque to the wing skeleton. The torque generated by the primaries travels through the wrist to the end of the radius-ulna, and the torque generated by the secondaries travels through the humerus to the shoulder. Therefore, in the above three joints (shoulder, elbow, and wrist), tendons control the contraction of muscles in different parts to promote the motion of different bones [17]. The shoulder mainly executes flapping and swing, and the elbow and wrist mainly execute stretching and twisting, respectively, which form the “twist—swing” motion. The tendons also increase the twist angle by controlling the secondaries to curve downward. Although the bionic structure of a bird wing demonstrates an ideal mechanism for realizing twist, and swing, it depends upon the distributed muscle tendon to implement the motion that is not feasible to mimic by the mechanical mechanism [18].

The movement and posture of birds’ wings are controlled by the internal skeleton, so many scholars have studied the skeleton of bird wings [19–21]. The layout of the wing skeleton is similar to that of most vertebrates: the distal humerus joins the proximal ends of the radius and ulna. The radius and ulna connect the distal ends to the carpal bone, which connects proximally to the metacarpal bone [22]. However, the radial and ulna of a bird wing are distributed side by side, a skeletal arrangement that produces a "drawing parallels" mechanism similar to the four-bar mechanism [23]. The phenomenon, first documented by Bergman in an 1839 autopsy, is that the wing elbow and wrist joints are coupled to each other, and the wrist joints contract as the elbow joints contract. Subsequently, Fisher verified this mechanism through two sets of experiments [24]. In the first experiment, the flexor and extensor muscles of the metacarpal bones were cut off. However, the wings of the pigeons were able to extend and contract, and they could fly normally, but their flight became somewhat clumsy. In the second experiment, pigeons were able to fly by shortening their radial bones by 5 millimeters (the original radius was 48 mm long), but when the flexors and extensors of the metacarpal bones were further removed, the wings were unable to contract and fly. The experimental results preliminarily show that pigeons can realize wing expansion and contraction without the flexor and extensor muscles of the metacarpal bones. Vazquez analyzed the anatomy of pigeons and concluded that the extensor primaris radiales could passively pull the wings to extend during wing extension [1]. In the process of wings contraction, the flexor carpi ulnaris muscle can passively pull the wings to contract, that is, the linkage mechanism of wing bones is not only the function of the parallel distribution of the ulna and radius, but also the muscle contributes to the extension and contraction of birds’ wings [25].

The joint mechanism of bird wing bones has many functions. The first function is that the wrist joint of the wing contracts with the elbow joint, which can quickly change the shape of the wing. The second function is to limit wing expansion posture, reduce wing joint degrees of freedom, and make flight control easier [26]. The third function is to reduce the amount of muscle needed to control the wrist joint, making the flapping wing less powerful during flight. Burgess took the seagull as the research object to establish a theoretical model: assuming that the wings have no linkage mechanism, muscles similar to biceps and triceps were added to the wrist joint to realize the telescopic posture of the wrist joint, and then the flight power of birds was calculated. The flight power of birds is calculated when the wing linkage mechanism is assumed. The results show that the reduction of wrist muscles reduces the total flight power by 0.6%-1.2% [27].

In conclusion, the wing skeleton has a four-bar linkage mechanism, which enables the wing to move and deform flexibly. However, there are few studies on the movement posture of bird wing bones, which need to be further studied and improved.

## Movement of the wing bones

### CT scan experiments

Computed tomography (CT) scan experiments of a Falco Peregrinus were conducted because it is one of the world’s fastest birds, reaching speeds of 320 km/h before striking its prey. they hunt by clenched talons and kill by impact using their high speed and manoeuvrability. A carcass of Falco Peregrinus, dead and frozen for a long time, was donated by the Zhejiang Museum of Natural History. significant information about forelimb structure and function was obtained from the study of the specimen. The falcon had no signs of emaciation, decomposition, or other trauma-induced muscle abnormalities.

**Fig 2:**
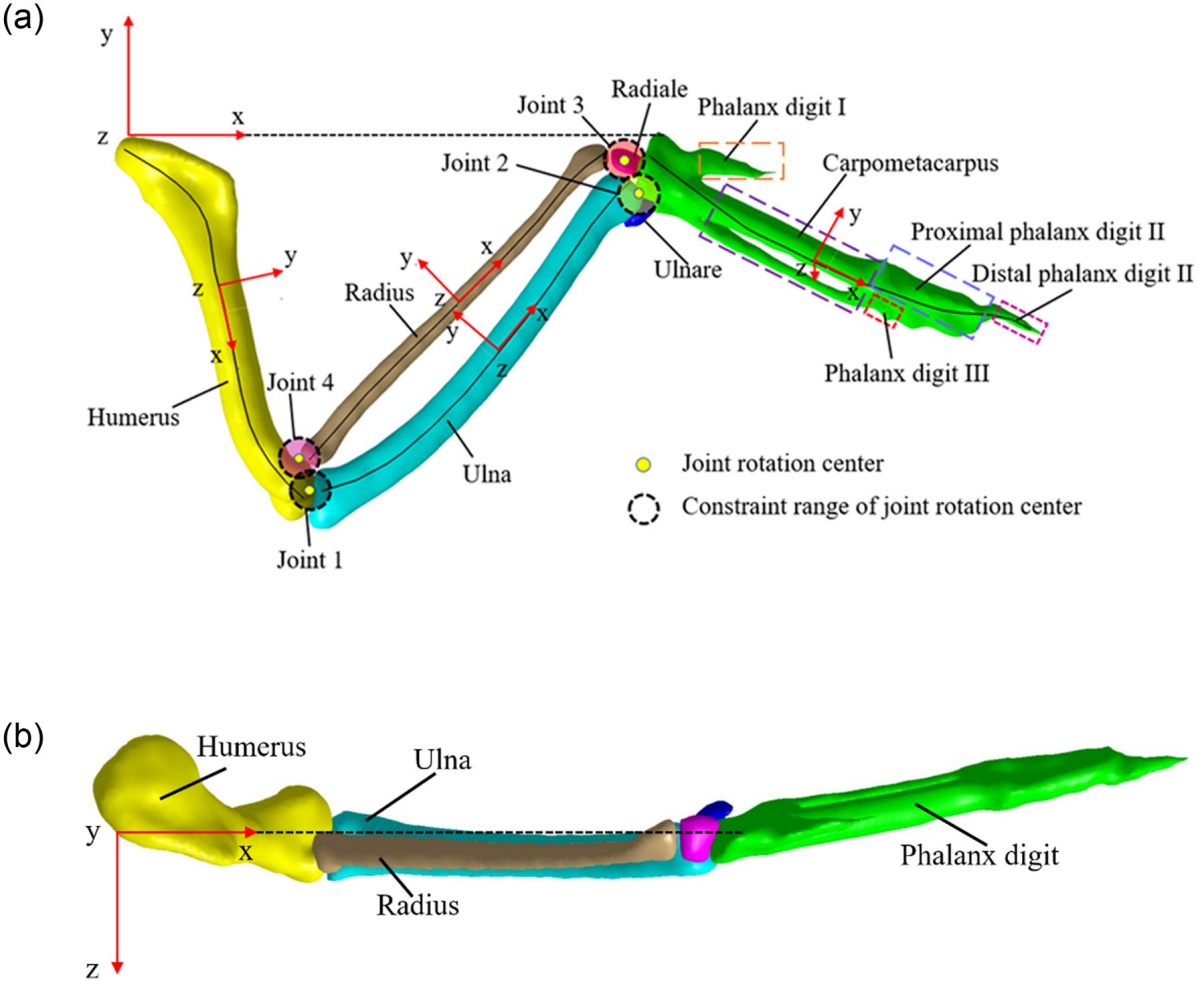
Forelimb elements and inertial coordinate of Falco Peregrinus. (a) in the dorsal view with scanned skeleton, (b)Vertical view.

Thus, a CT scan of the falcon wing was performed to show the relative positions of all ten bones, as shown in Fig 2(a). Like other avian species, the bones of the falcon wing comprise the humerus, ulna, radius, carpus, metacarpus, and digits. The skeleton of the wing is characterized by simplifications and reductions in the form of ankylosis, especially at the tip of the limb [28]. Similar to mammals, the humerus forms the skeleton of the brachium, and the ulna and radius form the skeleton of the antebrachium. Conversely, only the ulnar carpal (ulnare) and radial carpal (radiale) bones, originating from the proximal row of the carpal bones, remain in the falcon [29]. In contrast to humans, the metacarpals of the falcon wrist degenerate into major metacarpal and minor metacarpal and are then incorporated into the metacarpus. In addition, digit bones are considerably reduced. digit I (alular digit) and digit III possess only one phalanx, while digit II has two phalanges [30]. As a result, the elbow and carpus function as mutually dependent revolute joint joints and can therefore be extended or flexed in tandem.

### Wing skeleton coordinate system and fitting functions

Establish the global coordinate system of the wing skeleton: as shown in Fig 2(b), The humerus, radius, ulna, and carpometacarpus lie primarily in the same plane when the wing is fully extended, we define it as the XY plane. The Head of the proximal humerus is the origin of the global coordinate system. The line connecting the proximal head of the humerus to the proximal head of the metacarpal is defined as the X-axis. The y-axis is perpendicular to the x-axis, and the z-axis is arranged perpendicular to this plane, the direction of the Z-axis is opposite to the direction of gravity.

Establish the local coordinate system of the wing bones: as shown in Fig 2(a), The origin of the local coordinate system of each skeleton is located at the midpoint of the centerline of the wing skeleton. Since the humerus, ulna, and radius are myopic in the same plane when the wings are fully extended. The XY plane of the local coordinate system of the humerus-ulna-radius is defined to be parallel to the XY plane of the global coordinate system. The XY plane of the metacarpal is defined as plane1 of the metacarpal. The axis perpendicular to the XY plane of the skeleton is defined as the Z-axis. The projection curve of the tangent direction of the midpoint of the bone centerline in the XY plane is defined as the X-axis. The Y-axis is perpendicular to the x and z axes. The posture of the wing skeleton is represented by the ZYX Euler Angle. The rotation of the skeleton about the Z-axis is the yaw angle, the rotation about the Y-axis is the pitch angle, and the rotation about the X-axis is the roll angle.

When the skeleton is transformed from the fully extended posture to another posture under the global coordinate system, the rotation matrix and transformation are defined as *A*_*p_b*_ and *Tran*_*p_b*_, respectively. Then the transformed point can be calculated:

$${cloudnew}_{p\_b}=A_{p\_b}{cloud}_{extension\_b}+{Tran}_{p\_b}$$ (1)

Where the subscript p represents the extension, half-extension, and flexion postures of the skeleton. Subscript b represents each bone, such as the ulna, radius, metacarpal, and humerus. *A*_*p_b*_ = (*R*_*Z*_ (*α*) *R*_*y*_ (*β*) *Rx* (*γ*))_*p_b*_ is the ZYX Euler Angle rotation matrix, which represents the rotation matrix of bone *b* with full extension posture transformed into *p* posture under the global coordinate system. *Tran*_*p_b*_ is the translation operation. *cloud*_*p_b*_ represents the original points before transformation, while *cloudnew*_*p_b*_ represents the points after transformation.

The Eq (2) were used to calculate the fitting residual *resid*_*p_b*_ between the transformed point cloud *resid*_*p_b*_ and the original point cloud *cloud*_*p_b*_, and *resid*_*p_b*_ was used to judge the degree of coincidence between the two clouds.

$${resid}_{p\_b}\left({cloud}_{p\_b},{cloudnew}_{p\_b}\right)=\frac{1}{n1}\sum_{i=1}^{n1}{min}_{j=1}^{n2}\left(\left\|X_{i}-{Xnew}_{j}\right\|\right)$$ (2)

Where *X*_*i*_ and *n*1 is the location and point number of *cloud*_*p_b*_ respectively. *Xnew*_*j*_ and *n*2 is the location and point number of the *cloudnew*_*p_b*_ respectively. ‖∙‖ is the Euler norm. The minimum value *resid*_*p_b*_ is obtained for each point of the original skeleton and finds the average of all the minimum values. Due to the irregular shape of bones, the smaller the fitting residual *resid*_*p_b*_, the higher the degree of coincidence of bones.

Obviously, the residual is a nonlinear function of both rotation Euler angle *Euler*_*p_b*_ and displacement *Tran*_*p_b*_ when the full-extended skeleton is transformed into another posture. The fitting residual *resid*_*p_b*_ between the new skeleton point cloud and the initial skeleton point cloud can be calculated using Eq (3).

$${resid}_{p\_b}=function\left({Euler}_{p\_b},{Tran}_{p\_b}\right)$$ (3)

In this paper, the wing skeleton is simplified into a spatial four-bar mechanism, and the degree of reversion of the four-bar mechanism to the telescopic posture of the wing skeleton is calculated. In this chapter, the joint types of the wing skeleton are calculated and the joint types of the four-bar mechanism are determined. The bone fitting function and joint constraint equation of the four joint constraints are shown in Eqs (4) and (5). Eq (4) calculates the mean bone fitting residual *resid* from all bone transformations from the full extended pose to all other poses. Secondly, the joint constraint equation of (*n*_*p*_ − 1)*n*_*j*_ was established, as shown in Eq (5). The global optimization function Glob-al-Search was used to optimize the bone fitting function with four joint constraints (Eq (4)), and the mean bone fitting residual *resid*, joint rotation center, and rotation axis of the four-bar mechanism were obtained. By comparing the mean value of the bone fitting residual of the four-bar mechanism with that of the unconstrained condition, the reappearance degree of the telescopic posture of the wing skeleton of different types of four-bar mechanisms was analyzed. Among the four bone fitting methods for joint constraint conditions, the initial value of the joint rotation center and constraint range described above were also used.

$$resid=\frac{1}{\left(n_{p}-1\right)\left(n_{b}-1\right)}\sum_{b-1}\sum_{p-1}{resid}_{p\_b}$$ (4)

$$\left(n_{p}-1\right)n_{j}\left\{\begin{array}{c} A_{p\_b}X_{b\_b^{\prime }}+Tran_{p\_b}=A_{p\_b^{\prime }}X_{b\_b^{\prime }}+Tran_{p\_b^{\prime }}\\ or\left\{\begin{array}{c} A_{p\_b}X_{b\_b^{\prime }}+Tran_{p\_b}=A_{p\_b^{\prime }}X_{b\_b^{\prime }}+Tran_{p\_b^{\prime }}\\ {\vec{u}}_{b1}\cdot {\vec{u}}_{b^{\prime }3}=0\\ A_{p\_b}{\vec{u}}_{b1}\cdot A_{p\_b^{\prime }}{\vec{u}}_{b^{\prime }3}=0 \end{array}\right.\\ or\left\{\begin{array}{c} A_{p\_b}X_{b\_b^{\prime }}+Tran_{p\_b}=A_{p\_b^{\prime }}X_{b\_b^{\prime }}+Tran_{p\_b^{\prime }}\\ {\vec{u}}_{b1}\cdot {\vec{u}}_{b^{\prime }3}=0.{\vec{u}}_{b2}\cdot {\vec{u}}_{b^{\prime }3}=0.{\vec{u}}_{b1}\cdot {\vec{u}}_{b2}=0\\ A_{p\_b}{\vec{u}}_{b1}\cdot {A^{\prime }}_{p\_b^{\prime }}{\vec{u}}_{b^{\prime }3}=0.A_{p\_b}{\vec{u}}_{b2}\cdot A_{p\_b^{\prime }}{\vec{u}}_{b^{\prime }3}=0 \end{array}\right. \end{array}\right.$$ (5)

Where, *n*_*p*_ − 1 represents the number of postures except for full extension, *n*_*b*_ − 1 represents the number of wing bones except the humerus, and *n*_*j*_ represents the number of joints.

### Fitting result

In the current research, the unrestraint joints are used to study the actual rotations and rotations of the skeleton, where 3 DOFs of translation and 3 DOFs of rotations are included in the unrestraint-joint type. However, it’s not convenient to manufacture such a joint. In order to further calculate the degree to which the four-bar mechanism can reproduce the telescopic posture of the wing skeleton, two four-bar mechanisms are optimized in this paper. Detailly, each joint is simplified as one single ball joint resulting in an all-ball-joints model. And universal joints model is used to further reduce the DOFs of the wing skeleton.

The calculation results of the four-bar mechanisms are listed in Table 1. It is found that a minimal residual of 0.527mm is achieved in the unrestraint model. The larger the degree of freedom used, the smaller bone fitting residual will be achieved. On the other hand, the bone fitting residual of spherical joint constraint and universal joint constraint is the same. Therefore, the four-bar mechanism with four joints as universal joints (a full universal four-bar mechanism) is considered. It is implied that joints 2 and 3 cannot be reduced to ball joints according to the joint types of wings, therefore, the four-bar mechanism with all four joints as ball joints cannot accurately reproduce the telescopic posture of the wing bones.

**Table 1:** Residual of the four main bones.

Type	Half contraction Resid(mm)			Complete contraction Resid(mm)		
	Ulna	Humerus	Metacarpal	Ulna	Humerus	Metacarpal
Unrestraint	0.5097	0.4839	0.4991	0.5178	0.548	0.6035
All ball joints	0.5616	0.5	0.551	0.5754	0.5937	0.7956
Universal joints	0.5598	0.4995	0.5514	0.5759	0.5956	0.7954

**Fig 3:**
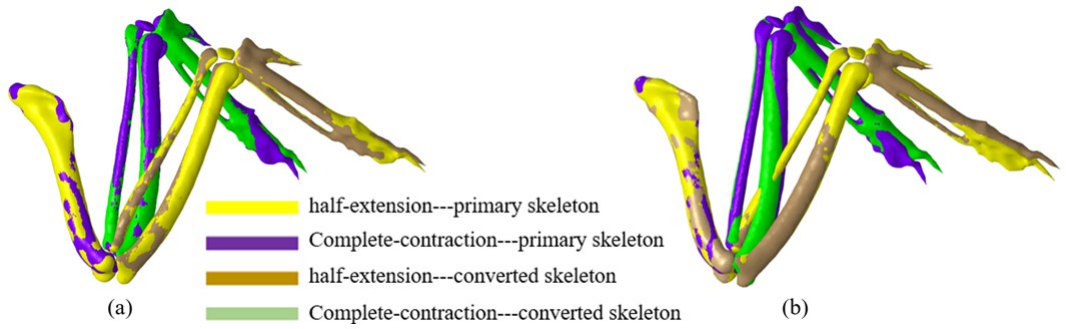
Skeletal fitting illustrates. (a) Global revolute joint four-bar mechanism (b) 1-DOF four-bar mechanism.

Compared with the calculation results without constraint conditions, the fitting deviations of each skeleton in both semi-contraction and full-contraction poses are increased. The mean bone fitting deviation of the global revolute joint four-bar mechanism and the full-universal joint four-bar mechanism is the same with a 0.5962 mm residual, and the relative deviation is 13.13% compared with the unconstrained condition. The bone fitting diagram of the global revolute joint four-bar mechanism is shown in Fig 3(a), indicating that although the four-bar mechanism cannot accurately reproduce the extension and contraction posture of the wing bones, the degree of coincidence is valuable.

In addition, the 4-bar mechanism with 1 degree of freedom is optimized. In the model, Joint 1 and Joint 2 are revolute joints, joint 3 is a universal joint, and joint 4 is a ball joint. The four-bar mechanism uses the ulna as the action bar, the radius and metacarpal as the driven bar, and the metacarpal contracts with the ulna. The mean bone fitting deviation of the 1-DOF four-bar mechanism is 0.9705 mm. The fitting result of the wing skeleton is illustrated in Fig 3(b), which can better reproduce the telescopic posture of the wing skeleton. In this paper, the 4-bar mechanism with 1 degree of freedom is used to design the bionic wing skeleton mechanism. The joint parameters of the 1-degree-of-freedom four-bar mechanism are listed in Table 2, which lists the joint rotation center, the joint rotation axis and the rotation angle of each joint. That is, the rotation angle of joint 1 is 15.54 degrees when it changes from a fully extended posture to a semi-extended posture, and 37.18 degrees is achieved when it changes from a fully extended posture to a fully contracted posture. The rotation angle of joint 2 was 22.84 degrees when the joint was converted from full extension to semi-extension, and 55.65 degrees was achieved when joint 2 was converted from full extension to full contraction.

**Table 2:** Joint parameters of the four-bar mechanism with one DOF.

Joint parameter	Center of joint rotation			Axis of joint rotation			Half-extension (°)	Full-extension (°)
	X(mm)	Y(mm)	Z(mm)	X(mm)	Y(mm)	Z(mm)		
Humerus and ulna	35.1385	-59.584	4.0225	-0.1037	-0.0838	0.9911	15.54	37.18
Ulna and metacarpal	89.9305	-7.229	7.5755	-0.022	-0.5246	0.8511	22.84	55.65
Radius and metacarpal	85.917	-0.6995	5.2835	0.7984	-0.1149	-0.591	-	-
Humerus and Radius	30.0535	-52.815	6.521	0.5989	0.0185	0.8007	-	-

In this section, three postures of wing skeletons are obtained through CT scanning experiments. An unconstrained bone fitting method is developed to obtain the posture data of the bones during the stretching process. The types of wing joints are studied by bone fit-ting with single joint constraints: Joints 1 and 4 could be simplified as universal joints or spherical joints. Joint 2 and joint 3 cannot be reduced to spherical joints, universal joints, and revolute joints. The mean bone fitting deviation of the two four-bar mechanisms is the same with a 0.5962 mm value, and the relative deviation of the two mechanisms is 13.13%. Although the four-bar mechanism cannot accurately reproduce the extension and contraction posture of the wing skeleton with a minimal fitting deviation, the relative deviation is endurable especially for bionic design. In addition, a 4-bar mechanism with 1 degree of freedom of extension/contraction is optimized to obtain a residual of 0.9705 mm. The motion posture of the wing skeleton has been reconstructed well. Thereafter, the bionic wing skeleton mechanism was designed which is discussed in the following section.

## Bionic design and experimental test

A bionic wing skeleton has been built using the optimized skeletal shape and joint data of the four-bar mechanism. Thereafter, the bionic wing skeleton mechanism was driven by the motor to achieve pitching and extending motions. The influence of the relative position of the connecting rope on the bionic skeleton was studied to find the appropriate end position. Finally, the motion accuracy of the bionic wing skeleton mechanism was verified by comparing the Adams simulation results with the actual measurement results.

### Bionic design of wing skeleton

**Fig 4:**
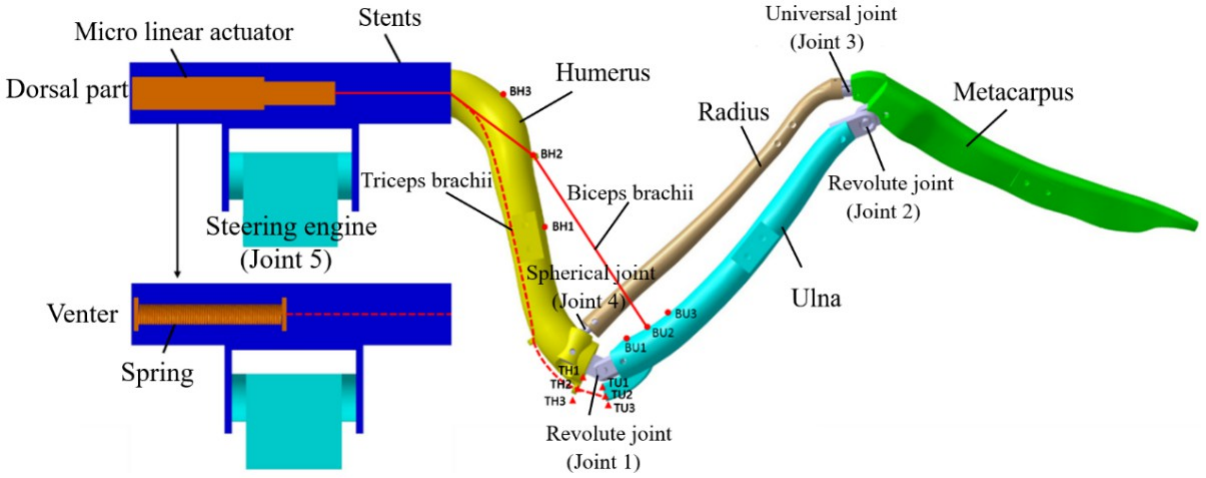
Bionic wing skeleton mechanism. The red solid line represents the bionic biceps brachii rope. The red dotted line represents the bionic triceps brachii rope.

In the bionic wing skeletal mechanism, a revolute joint (joint 1) was used to connect the bionic humerus and the bionic ulna, a spherical revolute joint (joint 4) was used to connect the bionic humerus and the bionic radius, a revolute joint (joint 2) was used to connect the bionic ulna and the bionic metacarpal, a universal joint (joint 3) was used to connect the bionic radius and the bionic metacarpal, and the shoulder joint was simplified with a steering engine. Thus, the main bionic bones together with the connected joints formed a 4-bar linkage mechanism with 2 degrees of freedom. The biceps brachii (rope 1) was used to connect the proximal end of the bionic ulna and the distal end of a linear motor across the bionic humerus, as shown in Fig 4. The triceps brachii (rope 2) was used to connect the proximal end of the bionic ulna and the distal end of a spring on the ventral side of the model across the elbow joint. The two ropes were a pair of antagonistic muscles similar to functions of the bicep’s brachialis and triceps brachialis of the natural bird wing.

We assume that the motion made by a bird’s wing is an extension and contraction motion in a two-dimensional plane, approximating the extension and contraction motion in a plane made by a human arm. When the linear motor pulls rope 1 to roll, rope 1 drives the bionic wing skeleton mechanism to extract. On the other hand, when the linear motor is extended, the spring rolls rope 2 passively to drive the bionic wing skeleton to extend. It can be seen that during the extracting motion, the rotation angle of the elbow joint changes from 0° to 37.18°, and the rotation angle of the wrist joint changes from 0° to 55.65°. A rotating joint (joint 5) is installed on the bionic wing skeleton mechanism, and the whole bionic wing skeleton can rotate by the steering engine to realize the pitch up and down movement. In current research, the rotation angle of joint 5 is limited between -30° to 30°. In the bionic wing skeleton mechanism, the linear motor is a miniature linear servo driver, model LA16 with a range of 0 *mm* to 16 *mm*, a maximum speed of 18 *mm*/*s*, a maximum tension of 70 *N*, and an accuracy of 0.2 *mm*. The stainless-steel spring model is 0.8 mm×8 mm×60 mm, and the stiffness coefficient of the spring is 0.187 *N*/*mm*, The RDS3120 high torque motor is selected for the steering gear, which can bear 20 *kg cm* torsions and rotate with a maximum angle of 180°. Use 0.75 mm PE wire was used for the rope. The bionic skeleton was printed by a 3D printer. The 3D printing material is PLA with a density of 1.3 *g*/*cm*^3^.

The end position of a rope on the bionic skeleton will affect the drive efficiency of the linear motor. In order to study this effect, three situations were set at each end. As shown in Fig 4, since the connection between the avian biceps brachii and the ulna is located at the proximal end of the ulna, three cases, BU1, BU2, and BU3, were set on the cranial side of the bionic ulna with a distance of 10 mm. As the connection between the avian bi-ceps brach ii and humerus is located at the proximal end of the humerus, three cases, BH1, BH2, and BH3, were set on the cranial side of the bionic humerus with a distance of 30 mm. Since the connection between the triceps brachii and the ulna was located at the olecranon of the ulna, three cases, TU1, TU2, and TU3, were set at the olecranon on the caudal side of the bionic ulna with a distance of 3 mm. Since the avian triceps brachii connects the ulna olecranon and the proximal humerus, three cases, TH1, TH2, and TH3 were set at the caudal side of the bionic humerus with a distance of 5 mm.

When the bionic wing skeleton mechanism is completely contracted, the stretching length and force of the linear motor reach the maximum value. Thereafter, Adams software was used to simulate 81 cases in total. The maximum expansion of the spring and maximum force of the linear motor were compared to select the appropriate endpoint position. The simulation process is illustrated as follows:

1. Joint type setting: Joints 1 and 2 are revolute joints, joint 3 is a universal joint, joint 4 is a spherical joint and joint 5 is a revolute joint;
2. Rope module: the rope module of Adams is used to simulate the rope actions. For example, one end of rope 1 is connected to the end of the bionic ulna, and the other end is connected to the linear motor. One end of the rope 2 is connected to the end of the bionic ulna, and the other end is connected to a spring through the end of the bionic humerus. A revolute joint is installed between bracket part 2 and the steering gear;
3. Initial parameter setting: the influence of the inertia force of objects is eliminated in the extracting motion, and the joint friction of the bionic wing skeleton mechanism is ignored, so that the force is only dependent on the endpoint position. The stiffness of the spring is set at 0.187 N/mm, and the initial preload of the spring is 4.5 N;
4. Motion setting: The motor rolls the rope to extend/extract the bionic wing skeleton mechanism at of speed of 8 mm/s;
5. Data processing: the force of the linear motor and displacement of the spring are calculated and compared for the 81 cases. The appropriate endpoint positions are optimized.

### Numerical experiments

**Fig 5:**
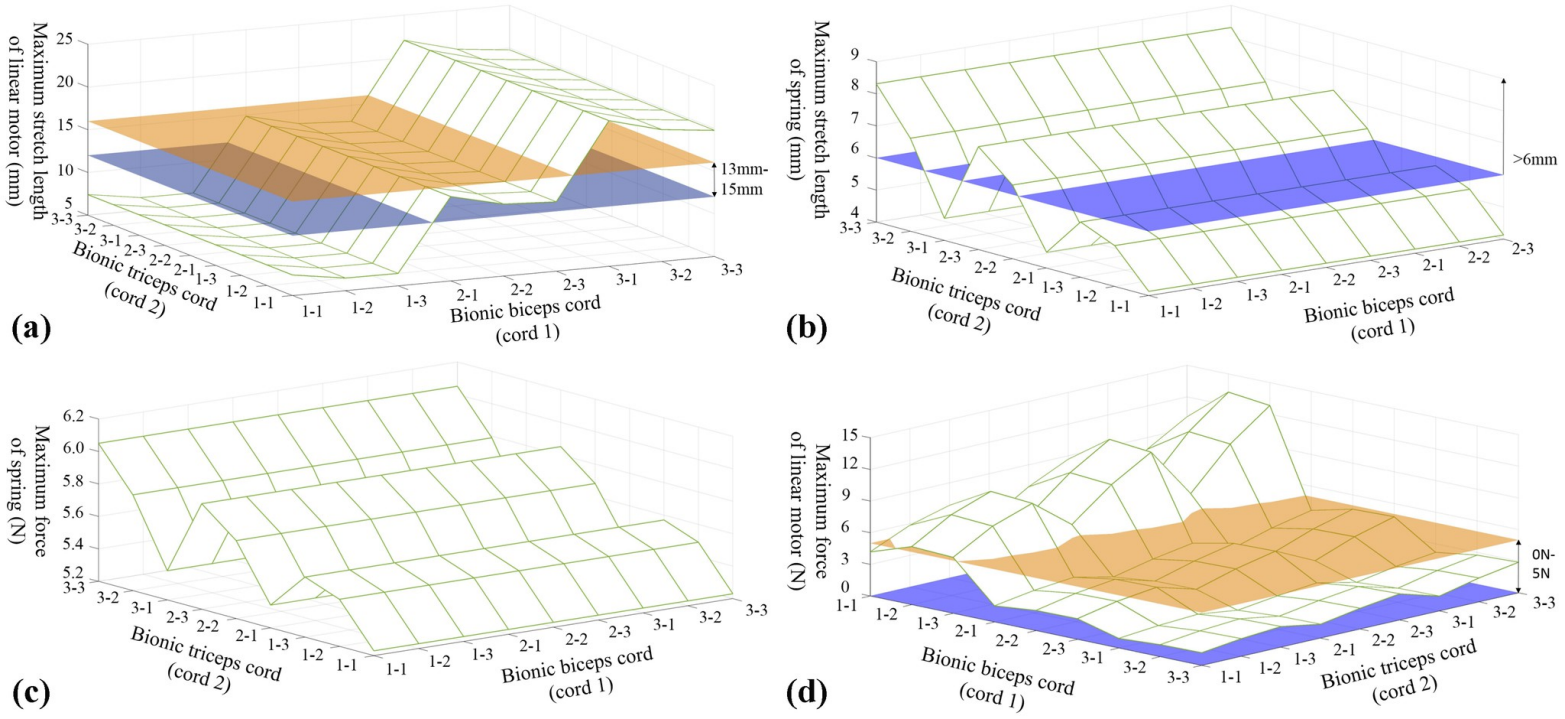
Measured motions of linear motor and spring. (a)The maximum length variation of linear motor (b)The maximum length variation of spring (c)The maximum force of spring (d)The maximum force of linear motor.

When the bionic wing skeleton mechanism is fully contracted, the stretch length of the linear motor reaches the maximum, as shown in Fig 5(a). It is shown that the maximum stretch length of the linear motor has nothing to do with the position of the end of rope 2. When the position of the cranial end of the bionic ulna changes from end 1 to end 3, the maximum stretch length of the linear motor increases significantly. When the position of the cranial end of the bionic humerus changed from end 1 to end 3, the maximum stretch length of the linear motor decreased slightly. It is implied that the cranial ends of the ulna and humerus formed a triangle with the revolute joint center point when the distance between the ulna cranial end and the revolute joint center (*length*_*ulna-hinge*_) is smaller than the distance between the humerus cranial and the revolute joint center (*length*_*humerus-hinge*_), he maximum stretch length of the linear motor increased against(*length*_*ulna-hinge*_). The position of the cranial end of the bionic ulna has a great influence on linear motor performances.

When the skeleton mechanism of the bionic wing is fully contracted, the stretch length of the spring reaches the maximum, as shown in Fig 5(b). When the connecting position moves from point 1 to point 3 on the ulna, the maximum stretch length of the spring increases gradually. When the bionic wing skeleton mechanism is completely contracted, the contraction force of the spring reaches the maximum value, as shown in Fig 5(c). At the same time, the driving force of the linear motor reaches the maximum value. In order to select a smaller maximum force for the linear motor, the constraint range of the maximum force of the linear motor is set at 0 N to 5 N, as shown in Fig 5(d). In a compromise with both the maximum stretch length and the maximum contract force, the 2–2 proximal ends are chosen for rop1 and rop2. When the bionic wing changes from full extension posture to full contraction posture, the linear motor stretches from 0 mm to 13.42 mm, and the driving force increases from 2 N to 3.16 N. The expansion capacity of the spring increases from 0 mm to 6.3 mm with the contracting force enhanced from 4.5 N to 5.68 N.

### Simulation experimental results

**Fig 6:**
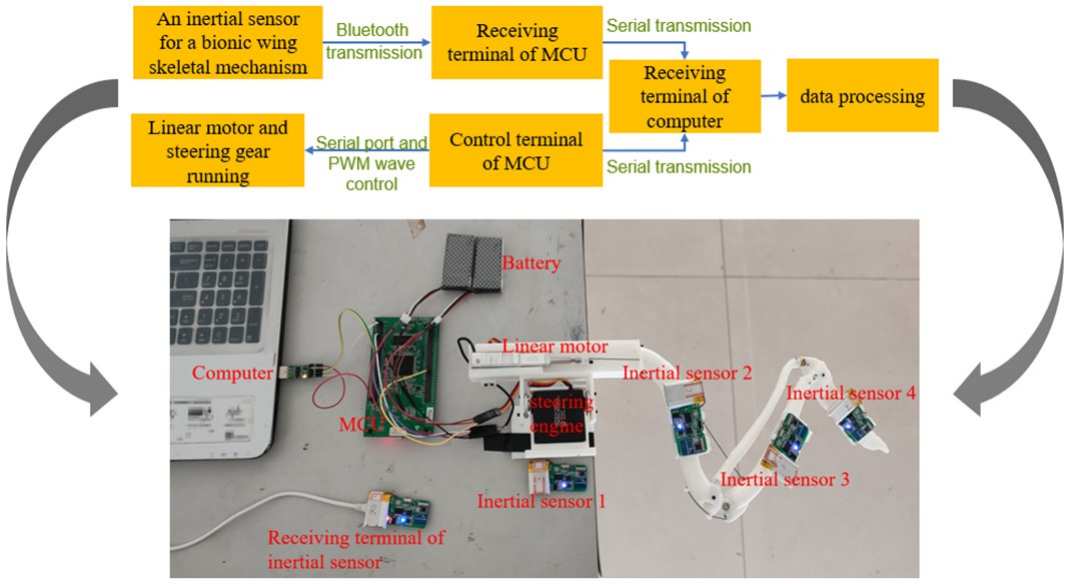
Experimental chart of bionic wing skeleton mechanism.

Fig 6 shows the experimental flow chart of motion measurement of the bionic wing skeletal mechanism and the experimental measurement chart. The experimental measurement is mainly divided into three parts:

1. In the motor control part: the single-chip microcomputer controls the linear motor to run at the speed of 8 mm/s to extract/extend the bionic wing skeleton, meanwhile it also the steering gear to pitch the wing skeleton
2. In the measurement part, MPU9250 inertial sensors are used to measure skeleton posture. The four inertial sensors are mounted on the steering gear, bionic humerus, bionic ulna, and bionic metacarpal. The inertial sensor transmits the attitude data of the skeleton to the computer through the Bluetooth module;
3. In the data processing part, the computer handles the transmitted data and calculates the relative rotations between neighboring bones.

In the data processing part, the quaternion of each bionic bone in the local coordinate system was collected at the initial time and the current time. Then calculate the relative quaternion between the time t and the initial time t_0_.

Assume that the quaternion of the inertial Sensor1 of the steering gear at time t is *p*_*S*1(*t*)_, *p*_*S*2(*t*)_ for Sensor2 on the humerus, *p*_*S*3(*t*)_ for the Sensor3 on the ulna, and *p*_*S*4(*t*)_ for the Sensor4 on the metacarpal. Thus, the relative quaternion between neighboring sensors can be calculated. Taking the bionic humerus as an example, the quaternions of the humerus’ inertial sensor under the steering gear’s coordinate can be calculated for both the initial and current time as shown in Eqs (6) and (7), where ⊗ is quaternion multiplication.


$$p_{S1(t)\_S2(t)}=p_{s1(t)}^{-1}⊗p_{S2(t)}$$ (6)

$$p_{S1(0)\_S2(0)}=p_{s1(0)}^{-1}⊗p_{S2(0)}$$ (7)

Assume the relative quaternion between *p*_*S*1(*t*)___*S*2(*t*)_ and *p*_*S*1(0)___*S*2(0)_ is $p_{S1(t)\_S2(t)}^{'}$ Calculate the relative quaternion between the current time and the initial time, as shown in Eq (8).


$$p_{S1(t)\_S2(t)}^{'}=p_{S1(t)\_S2(t)}⊗p_{s1(0)\_s2(0)}^{-1}$$ (8)

Similarly, the relative quaternions of the ulna and metacarpal are calculated using Eqs (9) and (10). Where $p_{S1(t)\_S2(t)}^{'},\;p_{S2(t)\_S3(t)}^{'}$ and $p_{S3(t)\_S4(t)}^{'}$ represent the relative rotation angles of joint 5, joint 1 and joint 2 at the current time.


$$p_{S2(t)\_S3(t)}^{'}=p_{S2(t)\_S3(t)}⊗p_{s2(0)\_s3(0)}^{-1}$$ (9)

$$p_{S3(t)\_S4(t)}^{'}=p_{S3(t)\_S4(t)}⊗p_{s3(0)\_s4(0)}^{-1}$$ (10)

**Fig 7:**
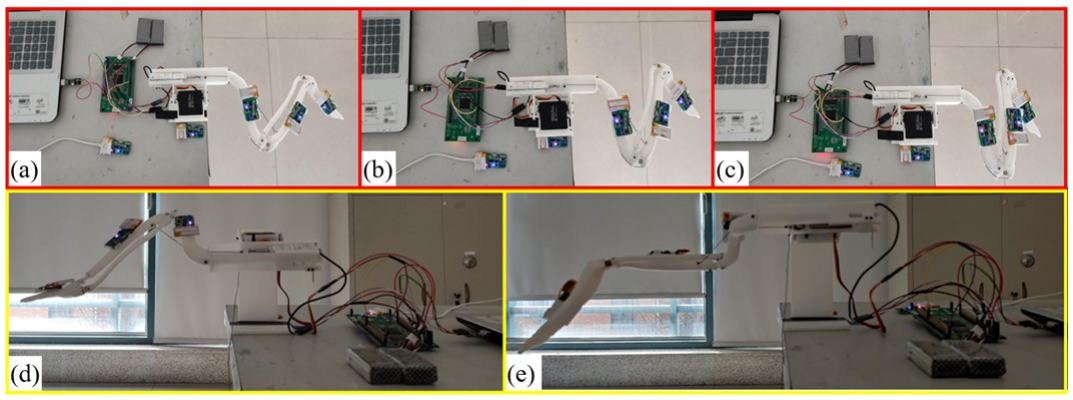
The attitudes of bionic wing skeleton mechanism. (a)extension (b)half-extension (c) flexion (d)pitch down (e)pitch up.

The bionic wing skeleton can morph under the control of the single-chip microcomputer. The full extension, half extension, and full contraction posture of the bionic wing skeleton mechanism respectively are shown in Fig 7(a)–7(c), and downward and upward postures are shown in Fig 7(d) and 7(e).

**Fig 8:**
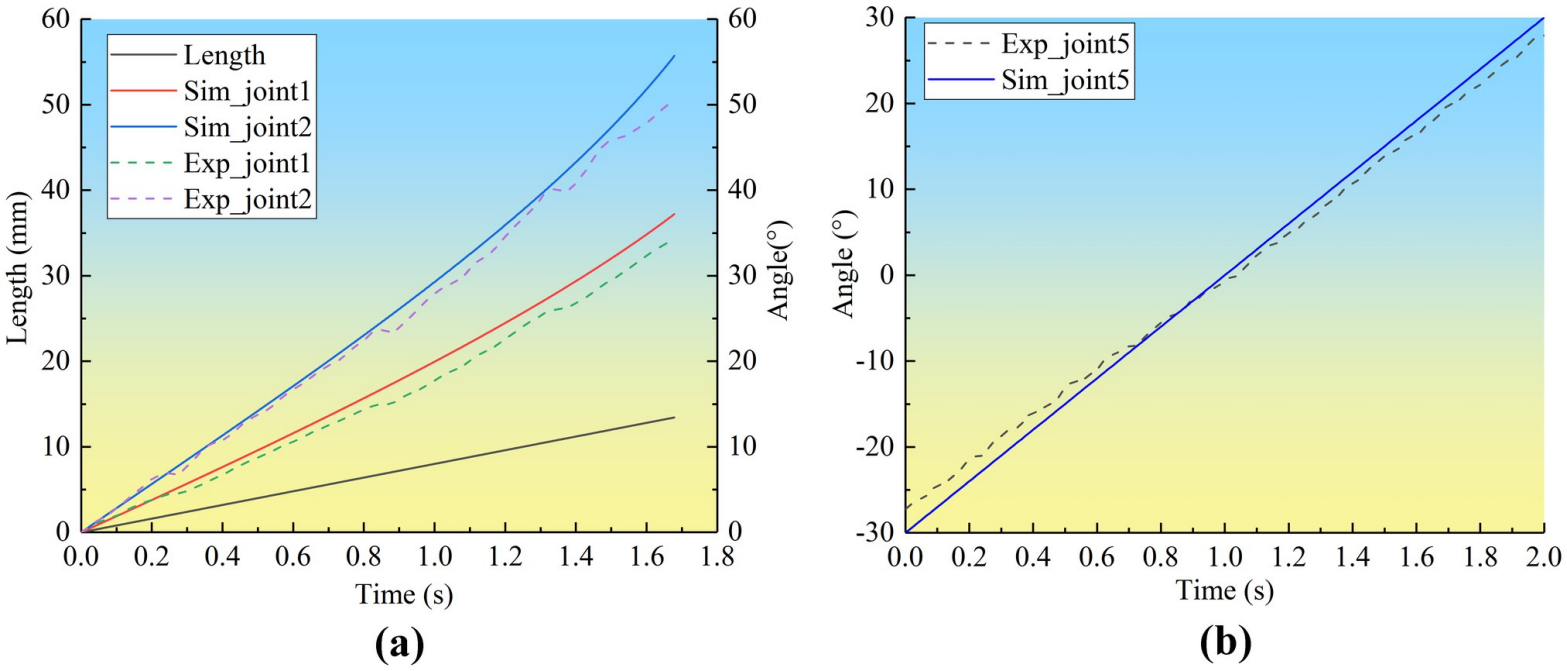
Comparison between experimental result and simulation results. (a) rotation angle of joint 1 and joint 2, (b) rotation angle of joint 5.

During extension, the rotating angles of each bone are collected in real-time. The experimental measurement results and simulation results are compared in Fig 8. Some deviations between the experimental and the simulation results are found. It is shown that the maximum deviation of the rotation angle of joint 1 is 3.3°, while the maximum deviation of the rotation angle of joint 2 is 5.6°. And a maximum deviation of 2.8° is found for the rotation angle of joint 5. Regarding the reason for the discrepancy between the simulated data and the experimental data, we consider that it is due to the assembly deviation of the bionic mechanism model, and since the discrepancy is relatively small, we believe that the result is in line with the expectation, but we are willing to do further work on it in anticipation of completing the results of the simulated data with even smaller discrepancy between the simulated data and the experimental data in the future. Above all, the extension and pitching movements of the bionic wing skeleton mechanism are reconstructed with high fidelity.

**Fig 9:**
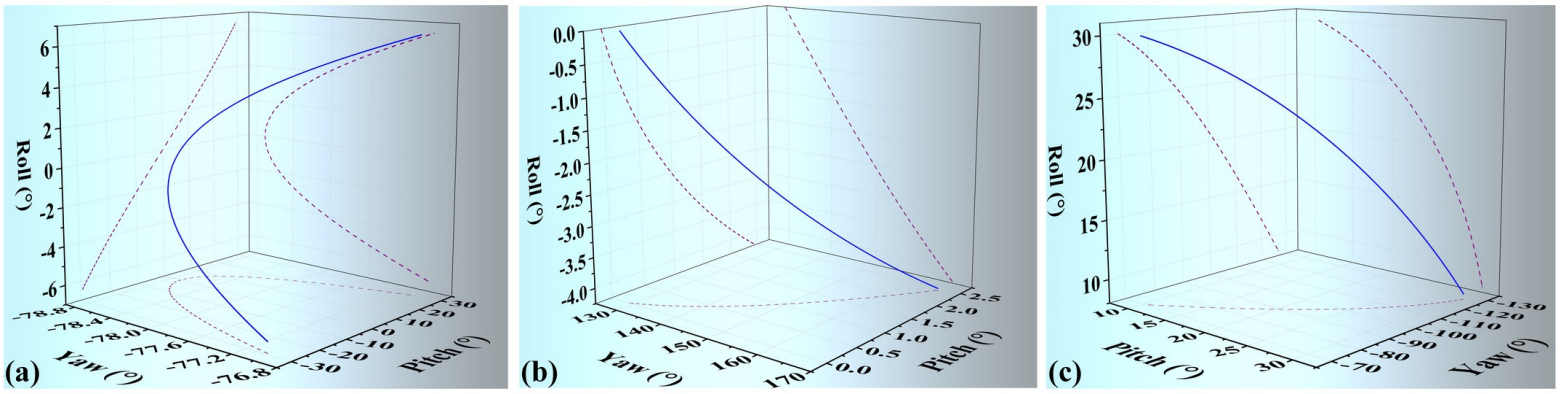
Posture data of bionic wing skeletal mechanism during movement. (a) The data of attitudes of the shoulder joint during mechanism pitch (b) The data of attitudes of the elbow joint during mechanism extension and flexion (c) The motion of wrist joint during mechanism extension and flexion.

The joint rotates during the stretching and pitching motions of the bionic wing skeleton mechanism. Euler angle of the shoulder joint is illustrated in Fig 9(a) during the pitching motion. It is shown that the yaw angle of the shoulder joint keeps almost the same, the roll angle varies from -6.44° to 6.45°, on the other hand, the pitch angle varies from -30° to 30°. It is implied that the pitch motion is the dominant motion of the shoulder joint in the current research. The Euler Angle of the elbow joint is illustrated in the Fig 9(b). The pitch angle and roll angle of the elbow joint remain unchanged, while the yaw angle changes from 129.3° to 166.09°. The Euler Angle of the wrist joint is illustrated in Fig 9(c). On the contrary to the shoulder and elbow joints, 3D motions of wrist joints are observed for the metacarpus. The yaw angle varies from -70.52° to -126.7° pitch angle varies from 10.53° to 30.63° and the roll Angle varies from 8.46° to 30.04°. Therefore, the metacarpus rotates in a three-dimensional manual, indicating the rotating axis of joint 2 is not consistent with the global coordinate axis which is validated by the axis of joint rotation listed in Table 2.

## Conclusion

The wing is one of the most important parts of the bird motor system and is the inspiration for the bionic wing design. For natural birds, wing shape is closely related to the size, shape and transformation angle of wing bones [1,31]. Therefore, it is of great reference value for the design of bionic variant wings to study the bone transformation law in wing morphing motions.

1. In this paper, the bone movement of a peregrine falcon was studied during wing extension and contraction motions. Based on the results, the wing skeleton was simplified as a space four-bar mechanism with one degree of freedom.
2. Motion similarity is judged and the residual is calculated, the bionic wing skeleton mechanism was designed according to the calculation results.

The observations of the current study can be a useful guide for the morphological analysis of birds and an enlightening inspiration for the bionic design of morphing aircraft in the future.

## Supporting information

S1 Data (XLS)
